# The Misdiagnosis and Underdiagnosis of Hepatic Encephalopathy

**DOI:** 10.14309/ctg.0000000000000784

**Published:** 2024-12-05

**Authors:** Patricia P. Bloom

**Affiliations:** 1 Division of Gastroenterology and Hepatology, Michigan Medicine, University of Michigan, Ann Arbor, Michigan, USA.

**Keywords:** cirrhosis, cognition, delirium, dementia, hepatic encephalopathy

## Abstract

Patients with cirrhosis are at risk of developing hepatic encephalopathy (HE), which can present with a wide range of symptoms, including confusion, lethargy, inappropriate behavior, and altered sleep patterns. In addition to HE, patients with cirrhosis are at risk of developing mild cognitive impairment, dementia, and delirium, which have features closely resembling HE. Given the similar presentation of these conditions, misdiagnosis can and does occur. Mild cognitive impairment is common in individuals aged 50 years and older and can progress to dementia in those affected. Dementia and HE are both characterized by sleep disturbance and cognitive dysfunction, thus differentiating these conditions can be difficult. Furthermore, delirium can disrupt sleep patterns, and liver disease is recognized as a risk factor for its development. As HE is a cirrhosis-related complication, determining if a patient has undiagnosed cirrhosis is critical, particularly given the large number of patients with asymptomatic, compensated cirrhosis. Separately, underdiagnosis of minimal HE can occur even in patients with diagnosed liver disease, related, in part, to lack of testing. Given the availability of effective therapies for managing symptoms and preventing future episodes, accurate diagnosis of HE is essential.

## INTRODUCTION

**Figure 1.**
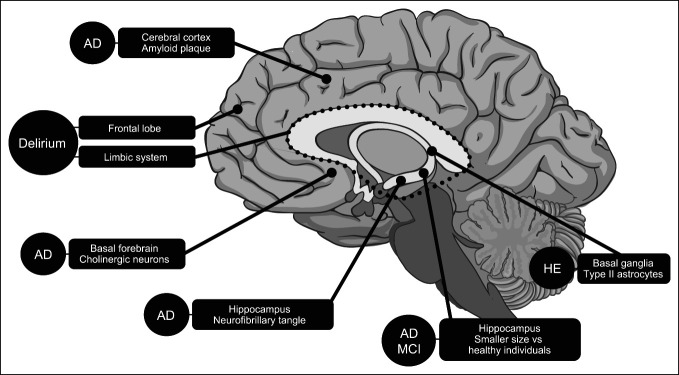
Regions of the brain affected by AD, HE, MCI, dementia, and delirium (7,10,11,13). AD, Alzheimer disease; HE, hepatic encephalopathy; MCI, mild cognitive impairment.

Hepatic encephalopathy (HE) is impaired brain function caused by liver insufficiency and/or portosystemic shunting (1) and has a wide range of presentations, from minimal (subtle cognitive impairment) to overt (observable clinical symptoms). Symptoms include confusion, lack of awareness, shortened attention span, personality changes, inappropriate behavior, sleep disturbances, and fatigue (Table 1) (1–13). Patients with cirrhosis are at risk of HE but often have comorbid conditions (e.g., alcohol use disorder, polypharmacy, diabetes) that increase the risk of other neurological disorders (14–16). Patients with cirrhosis are, on average, aged 60–62 years (17,18), which is important because older age is associated with an increased risk of mild cognitive impairment (MCI) and dementia development (19,20). Patients with cirrhosis are also at risk of delirium, defined as altered mental status characterized by inattention and fluctuating periods of consciousness (13,21). Thus, as patients with cirrhosis are at risk of developing HE, MCI, dementia, and delirium, which share certain clinical features (Table 1) despite distinct impacted brain regions (Figure 1), differential diagnosis of HE can be challenging (19,22–24). Furthermore, no neurological diagnostic tests have been validated to distinguish one of these conditions from another, which necessitates reliance on clinical symptoms and clinician judgment and can also complicate HE diagnosis.

**Table 1.** Summary of symptoms, diagnostic testing, and brain regions involved of 4 common neurological conditions in older adults and patients with cirrhosis

Condition	Brain regions involved	Clinical symptoms/classic manifestations	Validated neurological tests for diagnosis
HE (1–5)	Basal ganglia	Vary by severity, but include • Altered sleep patterns • Lethargy or apathy • Confusion • Personality changes • Inappropriate behavior • Asterixis • Coma	West Haven Criteria (minimal and grades 1–4) • Minimal HE ‐ No gold standard ‐ Tests include psychometric HE score, Animal Naming Test, EncephalApp Stroop Test, Sickness Impact Profile, and Inhibitory Control Test • Overt HE: ≥grade 2
Mild cognitive impairment (6–9)	Hippocampus is smaller in size compared with healthy individuals	Changes in cognitive function	DSM-5 criteria: • Modest decline in ≥1 cognitive domain • Decline in cognitive test performance • Insufficient to interfere with IADLs • Not from delirium or other disorders Brief cognitive assessment: • Calibrated to maximize sensitivity • Patients with positive results should undergo further evaluation • Tests include ACE-R, CERAD, CDT, Memory Alteration Test, MiniCog, MMSE, MoCA, and Qmci screen
Dementia (Alzheimer disease) (7,9–12)	Cerebral cortex (amyloid plaque) Hippocampus (neurofibrillary tangle within pyramidal neuron; smaller in size vs healthy individuals) Basal forebrain (cholinergic neurons)	Declining cognitive function, comprehension, judgment, memory, and self-control	DSM-5 criteria: • Substantial decline in ≥1 cognitive domain • Decline in cognitive test performance • Interferes with IADLs • Not exclusively from delirium or other disorder Cognitive assessments same as mild cognitive impairment
Delirium (13)	Frontal lobe Limbic system	Acute change in mental status, with altered consciousness and decrease in ability to focus coupled with cognitive changes or development of perceptual disturbance	DSM-5 criteria: • Disturbed attention, awareness, and cognition • Developed acutely from baseline • Not explained by neurocognitive disorder • Direct physiological consequence of another medical condition

ACE-R, Addenbrooke Cognitive Examination-Revised; CDT, Clock Drawing Test; CERAD, Consortium to Establish a Registry for Alzheimer's Disease; DSM-5, *Diagnostic and Statistical Manual of Mental Disorders, Fifth Edition*; HE, hepatic encephalopathy; IADL, instrumental activities of daily living; MMSE, Mini‐Mental State Examination; MoCA, Montreal Cognitive Assessment; Qmci, Quick Mild Cognitive Impairment.

**Figure 2.**
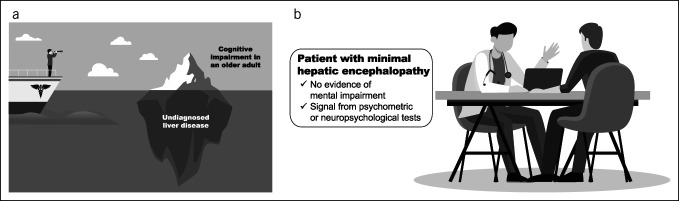
Main sources of underdiagnosis of hepatic encephalopathy: (**a**) undiagnosed liver disease and (**b**) challenges with diagnosing minimal hepatic encephalopathy.

Two main contributors can lead to misdiagnosis or underdiagnosis of HE (Figure 2). First, it is not uncommon for hepatologists to diagnose HE in patients who have long suffered from symptoms but have been misdiagnosed as having MCI, dementia, or delirium. This usually occurs when the patient lacks a liver disease diagnosis. Second, a source of underdiagnosed HE is minimal HE that is missed due to lack of testing. Minimal HE is subtle cognitive impairment that is detected by psychometric testing (1), which providers may not have the time or resources to routinely perform. Therefore, even if liver disease is known, minimal HE may go undiagnosed.

Addressing the problem of misdiagnosis and underdiagnosis of HE is critical in alleviating patient suffering, as HE interferes with activities of daily living (e.g., driving, employment), decreases quality of life (QOL), and is a considerable burden on caregivers (25–28). In a systematic review, HE was the only cirrhosis-related complication consistently (75% of studies) associated with impaired patient health-related QOL (28). In addition, patients with HE experience frequent hospitalizations and have an increased mortality risk (29,30). Even when controlling for other significant variables, severe HE during hospitalization independently predicted 30-day mortality (30). HE is largely treatable with pharmacologic therapies (i.e., lactulose and rifaximin), but making the correct diagnosis is a necessary prerequisite.

This review examines the possible misdiagnosis and underdiagnosis of HE. Factors that may contribute to misdiagnosis of HE as MCI, dementia, or delirium in patients with undiagnosed liver disease are reviewed, followed by factors contributing to underdiagnosis of minimal HE. Practical clinical considerations are provided for each scenario. The review concludes with opportunities for improving diagnosis of HE.

## METHODS

A systematic literature review was conducted using PubMed and included English-language articles published between January 1, 2003, and January 2, 2024. Studies eligible for inclusion were related to incidence and/or prevalence of dementia or HE and to diagnosis of dementia or HE. Search terms included “diagnosis,” “misdiagnosis,” “underdiagnosis,” “liver disease,” “liver cirrhosis,” “hepatic encephalopathy,” “alcohol consumption,” “alcohol use,” “delirium,” “dementia,” “memory disorder,” “cognition disorder,” “cognitive dysfunction,” and “cognitive impairment”.

A total of 6,071 articles were identified after removal of duplicates, 5,587 of which were excluded after title and abstract screening. Of 484 articles assessed for eligibility, 394 were excluded based on study design (n = 292 articles), patient population (n = 61), outcomes (n = 18), rigor (n = 10), comparator (n = 3), setting (n = 1), or otherwise irrelevant to the defined research questions (n = 9), yielding 90 relevant articles for inclusion.

## RESULTS

### Misdiagnosis in patients with undiagnosed liver disease

Patients with cirrhosis may not be formally diagnosed because they may have asymptomatic, largely compensated disease; normal liver biochemistries; and generally feel well (31). Indeed, more than half of patients with known cirrhosis have compensated disease (32,33), and there are an estimated 112 million global cases of compensated cirrhosis (2017) (34). One study noted that 56.3% of 5,118 deceased patients in the United Kingdom with cirrhosis were in the compensated stage at the time of death (33), and in another study, 30% of 105 Norwegian patients were not diagnosed with cirrhosis until autopsy (35). These data support the likelihood of a substantial proportion of patients with undiagnosed compensated cirrhosis globally. Patients with undiagnosed cirrhosis who develop altered cognitive function are likely to have only non-HE neurological disease considered in their differential diagnosis, as reviewed below.

#### Misdiagnosis as MCI or dementia.

MCI can precede dementia, both of which can impair daily activities (6,36). Prevalence of MCI varies widely (range, 2.5%–78.6%), with rates affected by the definition of MCI, diagnostic tools and scoring cutoffs used, and the population studied (37–41). Nevertheless, there appears to be a higher MCI prevalence in older individuals (i.e., age ≥50 years) (42,43). According to the US Aging, Demographics, and Memory Study, the estimated prevalence of cognitive impairment without dementia in individuals aged 71 years and older is 22.2% (approximately 5.4 million), with an annual rate of progression to dementia of approximately 12% (44). In the Adult Changes in Thought Study, using less vs more stringent criteria for establishing a diagnosis of MCI in adults aged 65 years and older without dementia, a prevalence range of 91.8% vs 11.3% was reported (45). Dementia appears to be slightly less common than MCI, with a dementia prevalence rate of 7.1% (meta-analysis of 9 European studies) to 8.3% (aged ≥65 years; US Medicare claims) (46,47). These values support the broader direction identified in other studies worldwide, albeit with some variation depending on diagnostic tests and the specific population studied (48–58).

##### Factors contributing to misdiagnosis:

A key reason for diagnostic mistakes is the considerable overlap in risk factors of MCI, dementia, and cirrhosis/HE. Common comorbid conditions in patients with dementia include alcohol use disorder (60.4%) and sleep apnea (28.4%), both of which play a role in cirrhosis pathogenesis, the latter by increased fibrosis caused by hypoxia (23,59–61). In multivariate analyses, dementia risk factors include impaired fasting glucose levels (5.6–6.9 mmol/L), hyperglycemia (>6.9 mmol/L), and treated diabetes—all risk factors of metabolic dysfunction-associated steatotic liver disease (MASLD)-related cirrhosis (50,62). A UK study of individuals aged 65–74 years in primary care reported that obesity was associated with long-term increased incidence of dementia (63). In another study, risk factors of dementia included diabetes, hypertension, and obesity—again, all risk factors of MASLD-related cirrhosis (56). A higher fatty liver index was associated with twice the odds of dementia (64). Even in the absence of cirrhosis, patients with MASLD may experience cognitive dysfunction (65) and/or display lower brain volume on imaging (66).

Another reason for HE misdiagnosis in a population with undiagnosed cirrhosis is the overlap in symptoms of dementia and HE, including physical frailty, cognitive dysfunction, and sleep disturbance (1,23). A Danish cohort study of individuals aged 50 years and older (N = 1,491,276) identified 2.8% with sleep disorders; of these, 3.0% had a subsequent diagnosis of dementia (67). An Italian study of patients aged 65 years and older found that a significantly greater percentage of patients with dementia vs healthy individuals had excessive daytime sleepiness (43.6% vs 29.0%; *P* = 0.008) (68). Altered sleep patterns are a common symptom of HE; thus, some patients with dementia with sleep issues may have undiagnosed cirrhosis and HE (69,70). In addition, a community study of adults in China aged 75–97 years (N = 1,585) reported that 14.2% had physical frailty and 34.1% had cognitive frailty, both common conditions in cirrhosis and HE (71). Given the overlap in physical frailty, cognitive dysfunction, and sleep disturbance, dementia and HE can be easily confused.

Hypothetically, it is also possible that HE is being misdiagnosed as dementia in areas with limited access to diagnostic testing and availability of specialists. A 2015 Danish registry study (N = 1,079,358 aged ≥65 years) reported regional differences in the diagnosis of dementia, with an age-standardized and sex-standardized national prevalence range of 2.5% to 3.6% across 5 regions (*P* < 0.0001) (72). Authors suggested that dementia may be underdiagnosed in some regions, potentially due to limited access to diagnostic work-up or quality of postdiagnosis management. Indeed, nationally 73.5% of patients were diagnosed by specialists, but there was significant regional variation in their diagnosis rate (range, 60.9%–90.5%; *P* < 0.0001) (72). Thus, the accurate diagnosis of dementia can be challenging if diagnostic tools and specialists are not available.

The diagnostic tests used to identify dementia do not distinguish between dementia and HE. Patients with dementia or HE both have low scores on the Mini-Mental State Examination (MMSE) (73,74). A meta-analysis of 32 studies examining the diagnostic accuracy of the MMSE for dementia in individuals aged 65 years and older in community and primary care settings found several cutoff points for MMSE with both a sensitivity and specificity >0.8, which indicates that most individuals with MMSE findings of dementia actually have dementia (75). Certainly, the precise cutoff point for MMSE influences sensitivity and specificity (76,77). Unfortunately, no MMSE cutoff point distinguishes dementia from HE (74,78).

Many older adults with cognitive impairment do not have a diagnosis of dementia, but many likely have underdiagnosed dementia, and some may have HE. In the US Health and Retirement Study of individuals aged 65 years and older with cognitive impairment consistent with dementia (N = 6,036,224), 91.4% were living with the condition but did not have a dementia-related diagnosis (79). A secondary follow-up analysis estimating prevalence by healthcare setting utilization (N = 5,841,453) indicated only a slight change, with 88.4% having not received a dementia-related diagnosis (80). Another study estimated >100,000 individuals within the US Medicare system (2008) had undiagnosed dementia (81). Analysis of linked data from the 2000–2014 Health and Retirement Survey and 1998–2015 Medicare claims data found a 90.2% adjusted undiagnosed rate for a memory-related diagnosis in individuals aged 66 years and older who, based on the Modified Telephone Interview of Cognitive Status score, had transitioned from cognitively normal to cognitively impaired (82). Additional information about these individuals with cognitive dysfunction but without a dementia diagnosis is lacking. With the median age-adjusted prevalence of cirrhosis in Europe of 833 per 100,000 individuals (83), HE is a possibility.

Some of the best evidence that undiagnosed cirrhosis may lead to misdiagnosis of HE as MCI or dementia comes from data in US veterans carrying a dementia diagnosis (84). Of 177,422 individuals with a diagnosis of dementia and without a cirrhosis diagnosis, 5.3% had an elevated Fibrosis-4 score (based on blood tests), which was suggestive of cirrhosis. In addition, 1 center conducted a detailed chart review of 89 patients with elevated Fibrosis-4 score and confirmed cirrhosis in most and even suspected HE in some, supporting the presence of undiagnosed cirrhosis and HE in patients with diagnosed dementia (84).

##### Practical clinical considerations:

Although it appears to be a distinct possibility that patients with HE are being misdiagnosed with dementia, patients with dementia are more likely to be hospitalized and therefore undergo additional medical scrutiny. Dementia has been associated with hospitalization (odds ratio [OR], 3.7) (46), and during these hospitalizations, there are opportunities for imaging and/or assessment of liver biochemistries that might ultimately lead to an HE diagnosis. In rural areas, the lack of access to dementia training and education and lack of specialists may contribute to an underdiagnosis of dementia (85). In the primary care setting, an ideal cognitive screening test would be brief, easy to administer and score, and have high sensitivity and specificity for detecting cognitive impairment (86). In the United States, the Medicare annual wellness visit includes a determination of whether a patient is cognitively impaired through a short (<5 minutes), validated instrument that can be administered by nonphysician clinical staff (87). Unfortunately, none of the currently available tests accurately differentiate dementia from HE.

#### Misdiagnosis as delirium.

Delirium has a range of characteristics that may fluctuate in presentation and severity, including acute onset of attention deficits, altered arousal, delusions, hallucinations, and mood changes (88,89). Delirium is common in both hospitalized older adults and those receiving outpatient care; a study of 311 hospitalized adults reported the estimated prevalence of delirium (by *Diagnostic and Statistical Manual of Mental Disorders, Fourth Edition*) to be 20.7%, and a study of 444 patients (mean age, 82.9 years) referred to an outpatient psychiatric center reported an estimated prevalence of delirium of 19.1% (88,90).

##### Factors contributing to misdiagnosis:

Misdiagnosis of HE as delirium in patients with undiagnosed liver disease may occur because of overlapping risk factors and symptoms. Delirium may be provoked by infection, dehydration, constipation, and toxin exposure, events that also can precipitate HE (21,91). In a German study (2012–2016), delirium (identified by* International Classification of Diseases, tenth revision* [*ICD-10*] coding) was significantly associated with a sleep disorder (OR, 1.6; *P* < 0.001) (92). As described above, sleep disturbance is a common manifestation of HE. Importantly, in a second German study of patients admitted to the intensive care unit (2017; n = 163), liver disease was found to be a risk factor of delirium (OR, 3.2; 95% confidence interval, 1.5–7.1; *P* = 0.03) (24).

##### Practical clinical considerations:

It is possible that a subset of patients with a delirium diagnosis could be experiencing HE secondary to undetected liver dysfunction. Given that both delirium and HE have a fluctuating presentation, determining whether a patient has underlying liver disease may be helpful to inform a diagnosis of HE (1,3,88,89). Improvement of symptoms following treatment with lactulose may be indicative of HE, and secondary prophylaxis with lactulose with or without rifaximin to reduce the risk of recurrence is recommended (1,3).

A clinical diagnosis is required to differentiate delirium, dementia, and HE. Liver biochemistries are a good starting point but can be normal in some patients with cirrhosis (31). Liver ultrasound can be considered, but should not be routinely recommended in patients with dementia. Serum ammonia testing is also not validated to differentiate dementia, delirium, and HE. In cases with unusual presentations or focal neurological signs, head imaging should be considered.

#### Misdiagnosis related to prior alcohol use disorder or other drug use.

Alcohol-related liver disease is increasing in the United States and globally (93,94). Alcohol use significantly increases the risk of cirrhosis decompensation, including the development of HE (*P* < 0.001) (95).

##### Factors contributing to misdiagnosis:

Alcohol use disorder can lead to liver disease and HE but can also separately induce nonhepatic neurological disorders, including acute alcohol withdrawal syndrome, alcohol-related dementia, and Wernicke's encephalopathy (96). Chronic alcohol use disorder, with or without comorbid cirrhosis, impairs cognitive function (96). Indeed, alcohol use increases the diagnosis rate of dementia in some populations (97). Therefore, a patient with a history of long-term alcohol use presenting with altered mental status could be suffering from alcohol intoxication, acute alcohol withdrawal, or HE (96). Furthermore, patients undergoing alcohol withdrawal can experience delirium that can be difficult to differentiate from HE (98).

##### Practical clinical considerations:

Unfortunately, there is no single diagnostic test that can distinguish nonhepatic alcohol-induced neurological disorders from HE; as with many of the above comparisons, a comprehensive evaluation of history, symptom trajectory, and alcohol biomarkers may distinguish the 2 etiologies. The presence of autonomic symptoms (e.g., sweating, nausea, palpitations), insomnia, rapid speech, anxiety, and whole-body tremors observed during delirium related to alcohol withdrawal can help distinguish it from HE, which presents with hypersomnia, slow or slurred speech, depressed mood, and tremors only visible in the hands (98,99). Although not diagnostic for HE, brain imaging is warranted in patients with alcohol-related cirrhosis if intracranial bleeding after a fall is suspected (3).

#### Underdiagnosis of minimal HE

Minimal HE is the lowest grade of HE severity, with subtle signs that can only be diagnosed by neuropsychiatric testing (1). However, minimal HE is associated with poor health-related QOL, risk of falls, motor vehicle accidents, substantial caregiver burden, and a high risk of overt HE development (27,100–102). The prevalence of minimal HE in patients with cirrhosis varies considerably by study, population, and the neuropsychiatric testing used to diagnose the condition. In different studies, patients with minimal HE were compared with healthy volunteers using several instruments, including the Psychometric HE Score (includes digit symbol test, number connection tests A and B, serial dotting test, and line tracing test), Stroop test, critical flicker frequency test, electroencephalogram (EEG) spectral analysis, trail-making test, and sickness impact profile (103–113). Using different tests, the prevalence of minimal HE ranged from 26% to 74% (103–113). A US study reported variation in the diagnosis of minimal HE, depending on the neuropsychological test used (113), and a systematic review (n = 20 studies) examining performance of point-of-care diagnostic tests found a wide variation and need for further calibration of testing cutoff values (5).

##### Factors contributing to underdiagnosis.

Minimal HE is a major source of HE underdiagnosis because it is challenging to diagnose in routine clinical care. Of note, minimal HE is associated with cognitive dysfunction and low scores on MMSE (114) and can be confused with MCI or dementia. Minimal HE is more prevalent in patients with alcohol-related cirrhosis compared with patients with MASLD based on the psychometric HE score (*P* = 0.007) (115). In an Italian study, diagnosis of minimal HE was more common in patients with alcohol-related cirrhosis (70%) than in patients with hepatitis C virus- (HCV-) related cirrhosis (27%) or nonalcohol/non-HCV-related cirrhosis (40%; *P* = 0.003) (103). Thus, HE should be considered in patients with alcohol-related cirrhosis with altered mental status; however, symptoms overlap with those of alcohol intoxication, alcohol withdrawal, intracranial bleeding, and other causes of mental status abnormalities (96).

##### Practical clinical considerations.

A blood-based minimal HE screening tool (using serum albumin and ammonia levels) has been developed and has undergone preliminary testing (116). Bedside measures were integrated to develop the MELD-Na-Activity-Chairstands-Quality of Life HE Score, which can predict the risk of overt HE development in patients with cirrhosis (117). As well, a tool that combines laboratory test results (i.e., low albumin levels, high bilirubin levels) and use of specific medications (i.e., beta-blocker, statin) was developed to help predict outpatients with cirrhosis at risk of HE (118). Thus, providers can assess patients with cirrhosis for HE risk using laboratory testing, reviewing medication use, and using relatively simple tests (e.g., chair stands).

EEG signatures of HE (e.g., triphasic waves, arrhythmic delta activity), dementia (e.g., increased delta power, reduced alpha and beta power), and alcohol withdrawal (e.g., hyperactive, fast trace) may be useful in differential diagnosis (99,119,120). A study of 150 patients, including 41 with minimal HE, found that the presence of cirrhosis significantly modulated the EEG signal, with increased theta power and decreased beta power in patients with cirrhosis compared with healthy controls; authors suggested that EEG may be helpful in detecting minimal HE in patients with cirrhosis (103). In a study of 65 patients (n = 21 with minimal HE) who underwent liver transplant, patients with a history of overt HE had significantly impaired psychometric and EEG performance before liver transplant and greater improvement 1 year after transplant compared with those without previous overt HE (121). Notably, however, cognitive impairment through neuropsychological assessment persisted across groups after transplantation (121); thus, HE should not be ruled out based on the history of liver transplant. Further research directly comparing EEG across conditions is needed to support the validity of EEG as a diagnostic tool. Development of these tools for minimal HE diagnosis is important, as an analysis of US claims data estimated that more than one-third (37.6%) of patients develop HE within 1 year of receiving a cirrhosis diagnosis (122), and a 5-year analysis of US nationwide inpatient sample data reported that HE-related hospitalizations made up 0.3% of all US hospitalizations (123).

## DISCUSSION

Cirrhosis and HE are underdiagnosed, and it is important to differentiate HE from other conditions with similar symptoms and clinical presentations (i.e., dementia, MCI, delirium). However, there are currently no validated instruments or screening tools that can differentiate HE from conditions with similar presentations, thus a clinical diagnosis is required. Estimating the degree of underdiagnosis and misdiagnosis of HE has been difficult, and future studies are needed. As HE is a cirrhosis-related complication, determining if a patient has undiagnosed liver disease is critical, given the estimated population with asymptomatic compensated cirrhosis. Indeed, patients may be asymptomatic from liver disease until they develop HE-related cognitive dysfunction. An accurate diagnosis of HE is important, as effective pharmacologic therapies are available for the treatment and reduction in risk of recurrence.

Primary care providers, geriatricians, and hospitalists are the front line of medical care for older patients with cognitive dysfunction. These providers should be aware of the increasing incidence of cirrhosis globally, especially from MASLD and alcohol-related liver disease. Cirrhosis and HE should be included in the differential diagnosis for patients presenting with changes in mental status. Development and validation of diagnostic tools and biomarkers to differentiate between HE and other forms of cognitive impairment is critical for improving diagnosis of HE.

## CONFLICTS OF INTEREST

**Guarantor of the article:** Patricia P. Bloom, MD.

**Specific author contributions:** P.P.B. was involved in the conceptualization of the manuscript, data curation, and drafting and revising the manuscript, including reviewing and editing the final draft for submission.

**Financial support:** Salix Pharmaceuticals provided funding and support for technical editorial and medical writing assistance. Salix Pharmaceuticals did not actively contribute to the content or have a role in the decision to submit, but did review the final copy. The author did not receive any compensation for development of this manuscript.

**Potential competing interests:** Dr. Bloom receives a research grant from Vedanta Biosciences and consults for Nexilico.
